# *Arthrobacter* sp. EpRS66 and *Arthrobacter* sp. EpRS71: Draft Genome Sequences from Two Bacteria Isolated from *Echinacea purpurea* Rhizospheric Soil

**DOI:** 10.3389/fmicb.2016.01417

**Published:** 2016-09-12

**Authors:** Luana Presta, Marco Fondi, Elena Perrin, Isabel Maida, Elisangela Miceli, Carolina Chiellini, Valentina Maggini, Patrizia Bogani, Vincenzo Di Pilato, Gian M. Rossolini, Alessio Mengoni, Renato Fani

**Affiliations:** ^1^Department of Biology, University of FlorenceFlorence, Italy; ^2^Department of Surgery and Translational Medicine, University of FlorenceFlorence, Italy; ^3^Department of Medical Biotechnologies, University of SienaSiena, Italy; ^4^Department of Experimental and Clinical Medicine, University of FlorenceFlorence, Italy; ^5^Clinical Microbiology and Virology Unit, Careggi University HospitalFlorence, Italy; ^6^Don Carlo Gnocchi FoundationFlorence, Italy

**Keywords:** endophyte, medicinal plant, plant growth promoting, antibiotics, resistance

## Introduction

One of the most promising, but still overlooked fields of microbiological research is represented by endophytic microorganisms, i.e., those organisms living in the tissues of host plants and/or in their rhizosphere (Rosenblueth and Martínez-Romero, 2006; Reinhold-Hurek and Hurek, 2011). These microbes are emerging as a new potential source of secondary metabolites and products, for exploitation in medicine, agriculture, and industry.

From a biotechnological perspective, a controlled (engineered) colonization of plant's tissues by some bacteria may be desirable because of their ability to produce a variety of plant growth promoting (PGP) molecules, spanning from siderophores, nitrogenases, hormones, and so on. In addition, host-microbe interaction confers indirect advantage to the plant, resulting from the inhibition activity exerted by the associated microbial community toward potential pathogens.

Moreover, in the field of medicine, there are examples of very well-known molecules derived by endophytes like antibiotics, antimycotics, and anticancer drugs. Also, it is still unknown if plant-associated bacteria may enhance (or be responsible for) some of the effects exerted by the extracts of medicinal plants (essential oils) (Kloepper and Ryu, 2006; Hardoim et al., 2008).

In this regard, in October 2012, in Casola Valsenio (Italy), a collection of microorganisms was isolated from both internal tissues and the rhizospheric soil of the medicinal plant *Echinacea purpurea*, as reported in Chiellini et al. (2014). Among others, two strains sampled from the rhizosphere and belonging to *Arthrobacter* species were identified, i.e., *Arthrobacter* sp. EpRS66 and *Arthrobacter* sp. EpRS71. Based on their antibiotic resistance profile, reported in Mengoni et al. (2014), and on further tests performed on these two strains, they were selected as good candidates for genome sequencing analysis. The last, will constitute a resource to deeply investigate their genomic features and to perform comparative genomics analysis. Moreover, in the aim of new drugs discovery, the genome sequence will facilitate the identification of putative genes responsible for the production of bioactive compounds.

## Materials and methods

### DNA extraction and sequencing

*Arthrobacter* sp. EpRS66 and *Arthrobacter* sp. EpRS71 strains were inoculated overnight on TSB medium at 30°C. Their genomic DNA was then extracted using the CTAB method (Perrin et al., 2015). Furthermore, the authenticity of the genomic DNA was confirmed by 16S RNA gene sequencing.

MiSeq sequencing system (Illumina Inc., San Diego, CA) was used to perform the whole genome shot-gun of the two organisms. The method used a 2 × 300 bp paired-end approach, which produced a genome coverage of 246.0 x for *Arthrobacter* sp. EpRS66 and 91x for *Arthrobacter* sp. EpRS71.

### Genome assembly and annotation

The quality of the obtained read pairs was evaluated by inspecting them with FastQC software package v. 0.52 (Kunde-Ramamoorthy et al., 2014). Poor quality bases were removed with StreamingTrim (Bacci et al., 2014). *De novo* assembly was performed by using SPAdes 3.5 software (Bankevich et al., 2012) with a k-mer length of 21, 33, and 55. After, those contigs with length inferior to 2000 bp were trimmed and the remaining (6 and 24 for *Arthrobacter* sp. EPRS66 and *Arthrobacter* sp. EPRS71, respectively) were launched in a multi-draft based analysis through MeDuSa scaffolder (Bosi et al., 2015), by using as references 5 *Arthrobacter* genomes retrieved at NCBI database (*Arthrobacter arilaitensis* Re117, *Arthrobacter* FB24, *Arthrobacter* Rue61a, *Arthrobacter aurescens* TC1, *Arthrobacter chlorophenolicus* A6).

Automated annotation of the two draft genome sequences has then been performed with NCBI Prokaryotic Genome Annotation Pipeline.

## Results

The last version of *Arthrobacter* sp. EpRS66 genome has a total length of 3,707,708 bp and embeds only 2 scaffolds (L50 equal to 1), with a mean G+C content of 59.27%. The annotation analysis identified a total of 3485 genes, of which 3383 have been annotated as coding DNA sequences (CDS), 29 as pseudogenes, 4 as rRNAs, 68 as tRNAs, and 1 as ncRNA.

The draft genome sequence of *Arthrobacter* sp. EpRS71 24 is 4,849,450 bp long and its contigs are set-up in 10 scaffolds (L50 equal to 1). The G+C content is 61.60%, a value slightly higher than the previous but still perfectly comparable with that of other *Arthrobacter* genomes sequenced so far. The annotation of *Arthrobacter* sp. EpRS71 genome revealed the presence of 4515 genes. This total amount includes 4379 proteins coding sequences, 71 pseudogenes, and 62 RNA (6 rRNAs, 55 tRNAs, 1 ncRNA) coding sequences.

Both genome sequences have been deposited at NCBI database and are available in both fasta and GenBank format; the GenBank accession number of *Arthrobacter* sp. EPRS66 is LNUU00000000 and the version reported in this work was named LNUU01000000; the GenBank accession number of *Arthrobacter* sp. EPRS71 is LNUV00000000 and the version reported in this work is LNUV01000000.

## Author contributions

This project was planned by RF and AM. The DNA extraction was performed by IM and EP. The DNA sequencing has been performed by GR and VD. The data processing has been performed by LP and MF. CC, VM, PB, and EM assisted substantially on the technical part of this work. All author contributed to writing and editing the present manuscript.

## Funding

This work was supported financially by Ente Cassa di Risparmio di Firenze (Project 2013.0657).

### Conflict of interest statement

The authors declare that the research was conducted in the absence of any commercial or financial relationships that could be construed as a potential conflict of interest.
